# Supplementary material for: Cytoarchitectonic, receptor distribution and functional connectivity analyses of the macaque frontal lobe
Source: eLife. 2023 Aug 14;12:e82850. doi: 10.7554/eLife.82850 (PMC10425179; doi:10.7554/eLife.82850)
Supplement: MDAR checklist [file elife-82850-mdarchecklist1.pdf]

Westbrook Centre
Milton Road
Cambridge CB4 1YG, UK
P 01223 855340
W elifesciences.org
T @elife

Materials Design Analysis Reporting (MDAR) Checklist for Authors

The MDAR framework establishes a minimum set of requirements in transparent reporting mainly applicable to studies in the life sciences.

eLife asks authors to **provide detailed information within their article** to facilitate the interpretation and replication of their work. Authors can also upload supporting materials to comply with relevant reporting guidelines for health-related research (see EQUATOR Network), life science research (see the BioSharing Information Resource), or animal research (see the ARRIVE Guidelines and the STRANGE Framework; for details, see *eLife*'s Journal Policies). Where applicable, authors should refer to any relevant reporting standards materials in this form.

For all that apply, please note **where in the article** the information is provided. Please note that we also collect information about data availability and ethics in the submission form.

Materials:

Newly created materials	Indicate where provided: section/figure legend	N/A
The manuscript includes a dedicated "materials availability statement" providing transparent disclosure about availability of newly created materials including details on how materials can be accessed and describing any restrictions on access.		*n/a Reason: Does not involve producing new material.*

Antibodies	Indicate where provided: section/figure legend	N/A
For commercial reagents, provide supplier name, catalogue number and RRID, if available.		*n/a Reason: Does not involve use of antibodies.*

DNA and RNA sequences	Indicate where provided: section/figure legend	N/A
Short novel DNA or RNA including primers, probes: Sequences should be included or deposited in a public repository.		*n/a Reason: Does not involve creation or use of DNA or RNA sequences.*

Cell materials	Indicate where provided: section/figure legend	N/A
Cell lines: Provide species information, strain. Provide accession number in repository OR supplier name, catalog number, clone number, OR RRID.		*n/a Reason: Does not involve use of cell lines.*
Primary cultures: Provide species, strain, sex of origin, genetic modification status.		*n/a Reason: Does not involve use of cell cultures.*

Experimental animals	Indicate where provided: section/figure legend	N/A
Laboratory animals or Model organisms: Provide species, strain, sex, age, genetic modification status. Provide accession number in repository OR supplier name, catalog number, clone number, OR RRID.	*Laboratory animals are provided with information: species, brain ID, sex, age in the Material and Methods section (lines 156-158, 162-164).*	
Animal observed in or captured from the field: Provide species, sex, and age where possible.		*n/a Reason: Our study did not involve the use of samples collected in the wild.*

Plants and microbes	Indicate where provided: section/figure legend	N/A
Plants: provide species and strain, ecotype and cultivar where relevant, unique accession number if available, and source (including location for collected wild specimens).		*n/a Reason: Our study is a neuroanatomical study of a non-human primate brain.*

Microbes: provide species and strain, unique accession number if available, and source.		*n/a Reason: Our study is a neuroanatomical study of a non-human primate brain.*

**Human research participants**	**Indicate where provided: section/figure legend) or state if these demographics were not collected**	**N/A**
If collected and within the bounds of privacy constraints report on age, sex, gender and ethnicity for all study participants.		*n/a Reason: Our study is a neuroanatomical study of a non-human primate brain.*

Design:

**Study protocol**	**Indicate where provided: section/figure legend**	**N/A**
If the study protocol has been pre-registered, provide DOI. For clinical trials, provide the trial registration number OR cite DOI.		*n/a Reason: Our study is not a clinical trial.*

**Laboratory protocol**	**Indicate where provided: section/figure legend**	**N/A**
Provide DOI OR other citation details if detailed step-by-step protocols are available.	*We used an observer-independent, quantitative method to identify cortical borders. For each area, we analyzed the distribution patterns of 14 different receptor types and functional connectivity. Our methods have been described in* ***Material and Methods*** *(for details, cyto- and receptor architectonic methods*	

	are previously described by Palomero-Gallagher and Zilles 2018 (doi:10.1016/B978-0-444-63639-3.00024-4); Zilles et al. 2002 (doi:10.1016/s0924-977x(02)00108-6), and for functional connectivity analyses see Xu et al. 2019 (doi:10.1016/j.bpsc.2019.02.005).	

**Experimental study design (statistics details) ***		
**For in vivo studies: State whether and how the following have been done**	**Indicate where provided: section/figure legend. If it could have been done, but was not, write "not done"**	**N/A**
Sample size determination		*n/a Reason: Our study is a neuroanatomical study done on postmortem brains.*
Randomisation		*n/a Reason: Our study is a neuroanatomical study done on postmortem brains.*
Blinding		*n/a Reason: Our study is a neuroanatomical study done on postmortem brains.*
Inclusion/exclusion criteria		*n/a Reason: Our study is a neuroanatomical study done on postmortem brains.*

Sample definition and in-laboratory replication	Indicate where provided: section/figure legend	N/A
State number of times the experiment was replicated in the laboratory.		*n/a Reason: Our study is a neuroanatomical study done on postmortem brains.*
Define whether data describe technical or biological replicates.		*n/a Reason: Our study is a neuroanatomical study done on postmortem brains.*

Ethics	Indicate where provided: section/submission form	N/A
Studies involving human participants: State details of authority granting ethics approval (IRB or equivalent committee(s), provide reference number for approval.		*n/a Reason: Our study is a neuroanatomical study of a non-human primate brain.*
Studies involving experimental animals: State details of authority granting ethics approval (IRB or equivalent committee(s), provide reference number for approval.	*Brains were obtained as a gift from professor Deepak N. Pandya and from Covance Laboratory (Münster) (See Material and Methods, lines 156-158, 162-164). All procedures described in this study had the approval of the Institutional Animal Care and Use Committee and complied with the NIH Guide for Care and Use of Laboratory Animals, and the European Communities Council Directive.*	

Studies involving specimen and field samples: State if relevant permits obtained, provide details of authority approving study; if none were required, explain why.		*n/a*<br>*Reason: Our study did not involve the use of samples collected in the wild.*

**Dual Use Research of Concern (DURC)**	**Indicate where provided: section/submission form**	**N/A**
If study is subject to dual use research of concern regulations, state the authority granting approval and reference number for the regulatory approval.		*n/a*<br>*Reason: Our study is not subject to dual use research.*

Analysis:

**Attrition**	**Indicate where provided: section/figure legend**	**N/A**
Describe whether exclusion criteria were pre-established. Report if sample or data points were omitted from analysis. If yes, report if this was due to attrition or intentional exclusion and provide justification.		*n/a*

**Statistics**	**Indicate where provided: section/figure legend**	**N/A**
Describe statistical tests used and justify choice of tests.	*Statistical analyses described in* ***Material and methods*** *section for receptor densities (lines 358-383), for functional connectivity (lines 385-422), and for receptor fingerprints (lines 424-451).*	

**Data availability**	**Indicate where provided: section/submission form**	**N/A**
For newly created and reused datasets, the manuscript includes a data availability statement that provides details for access (or notes restrictions on access).	*Data will be made publicly available via the EBRAINS platform and the BALSA neuroimaging site upon publication (lines 52-53; 1548-1550).*	

When newly created datasets are publicly available, provide accession number in repository OR DOI and licensing details where available.	*The files with the parcellation scheme will be available via EBRAINS platform of the Human Brain Project (https://search.kg.ebrains.eu/instances/Project/e39a0407-a98a-480e-9c63-4a2225ddfbe4) and the BALSA neuroimaging site (https//balsa.wustl.edu/XXXXX)*	
If reused data is publicly available provide accession number in repository OR DOI, OR URL, OR citation.	*All datasets used here for analysis are openly available sources from the recently established PRIME-DE (http://fcon_1000.projects.nitrc.org/indi/indiPRIME.html)*	

**Code availability**	**Indicate where provided: section/figure legend**	**N/A**
For any computer code/software/mathematical algorithms essential for replicating the main findings of the study, whether newly generated or re-used, the manuscript includes a data availability statement that provides details for access or notes restrictions.	*Codes used will be publicly available (lines 420-422).*	
Where newly generated code is publicly available, provide accession number in repository, OR DOI OR URL and licensing details where available. State any restrictions on code availability or accessibility.	*Code used for the implementation and visualization of the functional connectivity analysis will be publicly available (https://github.com/seanfw/macaque-pfc-func-conn).*	
If reused code is publicly available provide accession number in repository OR DOI OR URL, OR citation.		*n/a*

Reporting:

The MDAR framework recommends adoption of discipline-specific guidelines, established and endorsed through community initiatives.

**Adherence to community standards**	**Indicate where provided: section/figure legend**	**N/A**
State if relevant guidelines (e.g., ICMJE, MIBBI, ARRIVE, STRANGE) have been followed, and whether a checklist (e.g., CONSORT, PRISMA, ARRIVE) is provided with the manuscript.		*n/a*

---

* We provide the following guidance regarding transparent reporting and statistics; we also refer authors to

[Ten common statistical mistakes to watch out for when writing or reviewing a manuscript].

Sample-size estimation

- You should state whether an appropriate sample size was computed when the study was being designed
- You should state the statistical method of sample size computation and any required assumptions
- If no explicit power analysis was used, you should describe how you decided what sample (replicate) size (number) to use

Since this is a neuroanatomical study without any statistical hypothesis testing, this information does not apply to this submission. We note that sample size (multimodal imaging data from four monkeys) is determent by practical and ethical constraints on non-human primate studies.

Replicates

- You should report how often each experiment was performed
- You should include a definition of biological versus technical replication
- The data obtained should be provided and sufficient information should be provided to indicate the number of independent biological and/or technical replicates
- If you encountered any outliers, you should describe how these were handled
- Criteria for exclusion/inclusion of data should be clearly stated
- High-throughput sequence data should be uploaded before submission, with a private link for reviewers provided (these are available from both GEO and ArrayExpress)

We used an observer-independent, quantitative method to identification of cortical borders, this method has proven to be a powerful tool in brain mapping. Detailed description can be found in the ***Materials and Methods*** *section.*

We analyzed cyto- and receptor architecture (of 14 different receptor types), as well as the distribution of functional connectivity pattern, and various subdivisions of the prefrontal and orbital cortex were recognized consistently across all studied hemispheres, the position, extent and distribution patterns of receptors as well as cytoarchitectonic features are well comparable between individuals. Additionally, the parcellation scheme and newly defined areas were reviewed by an experienced anatomist (NPG).

Statistical reporting

- Statistical analysis methods should be described and justified
- Raw data should be presented in figures whenever informative to do so (typically when N per group is less than 10)
- For each experiment, you should identify the statistical tests used, exact values of N, definitions of center, methods of multiple test correction, and dispersion and precision measures (e.g., mean, median, SD, SEM, confidence intervals; and, for the major substantive results, a measure of effect size (e.g., Pearson's r, Cohen's d)
- Report exact p-values wherever possible alongside the summary statistics and 95% confidence intervals. These should be reported for all key questions and not only when the p-value is less than 0.05.

Since this is a neuroanatomical study without any statistical hypothesis testing, this information does not apply to this submission.

Group allocation

- Indicate how samples were allocated into experimental groups (in the case of clinical studies, please specify allocation to treatment method); if randomization was used, please also state if restricted randomization was applied
- Indicate if masking was used during group allocation, data collection and/or data analysis

Since we did not perform any group comparisons, this information does not apply to this submission.
